# CEA, EpCAM, αvβ6 and uPAR Expression in Rectal Cancer Patients with a Pathological Complete Response after Neoadjuvant Therapy

**DOI:** 10.3390/diagnostics11030516

**Published:** 2021-03-14

**Authors:** Daan Linders, Marion Deken, Maxime van der Valk, Willemieke Tummers, Shadhvi Bhairosingh, Dennis Schaap, Gesina van Lijnschoten, Elham Zonoobi, Peter Kuppen, Cornelis van de Velde, Alexander Vahrmeijer, Arantza Farina Sarasqueta, Cornelis Sier, Denise Hilling

**Affiliations:** 1 Department of Surgery, Leiden University Medical Center, 2333 ZA Leiden, The Netherlands; d.g.j.linders@lumc.nl (D.L.); m.m.deken@lumc.nl (M.D.); m.j.m.van_der_valk@lumc.nl (M.v.d.V.); w.s.f.j.tummers@lumc.nl (W.T.); s.bhairosingh@lumc.nl (S.B.); p.j.k.kuppen@lumc.nl (P.K.); c.j.h.van_de_velde@lumc.nl (C.v.d.V.); a.l.vahrmeijer@lumc.nl (A.V.); c.f.m.sier@lumc.nl (C.S.); 2 Department of Surgery, Catharina Hospital, 5623 EJ Eindhoven, The Netherlands; dennis.schaap@catharinaziekenhuis.nl; 3 Laboratory of Pathology, Stichting Pathology and Medical Microbiology, 5623 EJ Eindhoven, The Netherlands; i.van.lijnschoten@pamm.nl; 4 Edinburgh Molecular Imaging Ltd., Edinburgh EH16 4UX, UK; e.zonoobi@lumc.nl; 5 Department of Pathology, Leiden University Medical Center, 2333 ZA Leiden, The Netherlands; a.farina@amsterdamumc.nl; 6 Percuros BV, 2333 CL Leiden, The Netherlands; 7 Department of Surgical Oncology and Gastrointestinal Surgery, Erasmus MC Cancer Institute, University Medical Center Rotterdam, 3015 GD Rotterdam, The Netherlands

**Keywords:** CEA, EpCAM, αvβ6, uPAR, fluorescence imaging, complete response, rectal cancer, tumor targeted, response evaluation, preoperative chemo and radiotherapy

## Abstract

Rectal cancer patients with a complete response after neoadjuvant therapy can be monitored with a watch-and-wait strategy. However, regrowth rates indicate that identification of patients with a pathological complete response (pCR) remains challenging. Targeted near-infrared fluorescence endoscopy is a potential tool to improve response evaluation. Promising tumor targets include carcinoembryonic antigen (CEA), epithelial cell adhesion molecule (EpCAM), integrin αvβ6, and urokinase-type plasminogen activator receptor (uPAR). To investigate the applicability of these targets, we analyzed protein expression by immunohistochemistry and quantified these by a total immunostaining score (TIS) in tissue of rectal cancer patients with a pCR. CEA, EpCAM, αvβ6, and uPAR expression in the diagnostic biopsy was high (TIS > 6) in, respectively, 100%, 100%, 33%, and 46% of cases. CEA and EpCAM expressions were significantly higher in the diagnostic biopsy compared with the corresponding tumor bed (*p* < 0.01). CEA, EpCAM, αvβ6, and uPAR expressions were low (TIS < 6) in the tumor bed in, respectively, 93%, 95%, 85%, and 62.5% of cases. Immunohistochemical evaluation shows that CEA and EpCAM could be suitable targets for response evaluation after neoadjuvant treatment, since expression of these targets in the primary tumor bed is low compared with the diagnostic biopsy and adjacent pre-existent rectal mucosa in more than 90% of patients with a pCR.

## 1. Introduction

Curative-intent treatment of locally advanced rectal cancer consists of neoadjuvant chemoradiation and surgical resection by total mesorectal excision [1,2]. Fifteen to twenty percent of rectal cancer patients achieve a pathological complete response (pCR) after neoadjuvant (chemo)radiotherapy [3]. In these patients, an organ-preserving strategy of watch and wait (W&W) could have been applied to avoid major surgery and its potential complications [4,5,6,7,8,9]. However, correct preoperative identification of patients with a pCR after neoadjuvant therapy by conventional imaging techniques including magnetic resonance imaging (MRI) and endoscopy is challenging [10]. A local regrowth rate of up to 38% indicates that a clinical complete response (cCR) does not always correspond to a pCR [7]. In those cases, understaging of residual tumor occurred [4,5,7,9]. On the other hand, MRI is known to overstage disease due to the difficulty of distinguishing fibrosis and mucin lakes from residual tumor, leading to unnecessary surgery in about 15% of patients who turned out to have a pCR in the resection specimen [10,11]. Therefore, enhanced diagnostic imaging tools are needed to identify a pCR more accurately and hence select the right patients for a W&W strategy. 

Tumor-targeted near-infrared (NIR) fluorescence imaging is a promising technique that combines the administration of targeted fluorescence contrast agents with the use of NIR fluorescence light (700–900 nm). It allows for real-time optical imaging by selectively highlighting cells that express certain molecular targets. Tumor-targeted fluorescence imaging during endoscopy could help to identify patients with a pCR, thereby minimizing under- and overtreatment of rectal cancer patients. Accurate response evaluation using fluorescence endoscopy requires a target with tumor-specific expression. Preferably, the protein of choice has (1) an exclusive upregulation by tumor or tumor-related stromal cells compared with surrounding pre-existent mucosa, (2) expression that is not influenced by neoadjuvant therapy, and (3) no expression in the tumor bed if there is no residual tumor. Various cancer-associated cell membrane proteins are currently under extensive investigation for tumor-targeted fluorescence imaging [12,13]. Promising targets in rectal cancer include carcinoembryonic antigen-related cell adhesion molecule 5 (CEACAM5, from here on to be referred to as CEA), epithelial cell adhesion molecule (EpCAM), αvβ6 integrin, and urokinase-type plasminogen activator receptor (uPAR) [14,15,16,17]. 

The cell membrane-bound glycoprotein CEA is involved in the regulation of adhesion, growth, and differentiation of epithelial cells, and is overexpressed on tumor cells of various origins, including colorectal cancer [13]. CEA has already been used as a target for fluorescence contrast agents in preclinical studies and recent clinical phase I/II trials [15,18,19]. CEA-targeted NIR imaging during colorectal cancer surgery provides an enhanced macroscopic differentiation between tumor and normal tissue. On the microscopic level, the pattern of the fluorescence signal in colon cancer tissue is consistent with CEA expression as measured using immunohistochemistry [15]. EpCAM is a transmembrane glycoprotein that plays a role in cell-to-cell interactions and adhesions [20]. The expression of EpCAM is highly upregulated in nearly all epithelial malignancies, such as colorectal adenocarcinoma [21,22,23]. Preclinical studies have demonstrated the feasibility of an EpCAM-specific fluorescence contrast agent for tumor visualization [14,24,25]. Integrin αvβ6 is overexpressed by malignant epithelial cells and activated endothelial cells and mediates adhesion to the basement membrane [26]. Integrin αvβ6 is overexpressed in colorectal adenocarcinoma and its upregulation is associated with increased metastatic potential and reduced survival [27]. In a preclinical study, an αvβ6-specific fluorescence contrast agent was developed and validated for imaging of pancreatic ductal adenocarcinoma [17]. uPAR, the cell membrane-bound receptor of urokinase-type plasminogen activator, is involved in tissue remodeling, cell signaling, and proliferation, and is overexpressed in most colorectal adenocarcinomas, largely due to upregulation by tumor-associated stromal cells [28,29,30]. Preclinical research showed the potential use of different uPAR-targeted fluorescence contrast agents in NIR fluorescence tumor imaging [16,31,32]. A phase I clinical trial has demonstrated the safe use and feasibility of uPAR-targeted positron emission tomography in different cancer types [33].

Expression of CEA, EpCAM, αvβ6, and uPAR is known to be upregulated by the majority of rectal cancer (associated) cells [20,26,29,34]. Previous immunohistochemical data demonstrated that CEA and EpCAM expression in rectal cancer cells and adjacent pre-existent mucosa does not change after neoadjuvant therapy. It showed that these proteins are still overexpressed in patients with a partial or no response after neoadjuvant therapy [35]. These findings suggest CEA and EpCAM are suitable targets for accurate response evaluation after neoadjuvant therapy using fluorescence endoscopy. The current study is a continuation of this research to further evaluate which of the rectal cancer-associated membrane proteins CEA, EpCAM, αvβ6, and uPAR is the most useful indicator of absence of residual vital cancer cells after neoadjuvant therapy. To this end, we analyzed protein expression by immunohistochemistry in resected primary tumor beds and corresponding diagnostic biopsies of rectal cancer patients with a pathological complete response after neoadjuvant therapy and compared both. The protein expression levels in the tumor bed were also compared to adjacent pre-existent rectal mucosa.

## 2. Material and Methods

### 2.1. Human Rectal Cancer Tissue Samples

**Figure 1:**
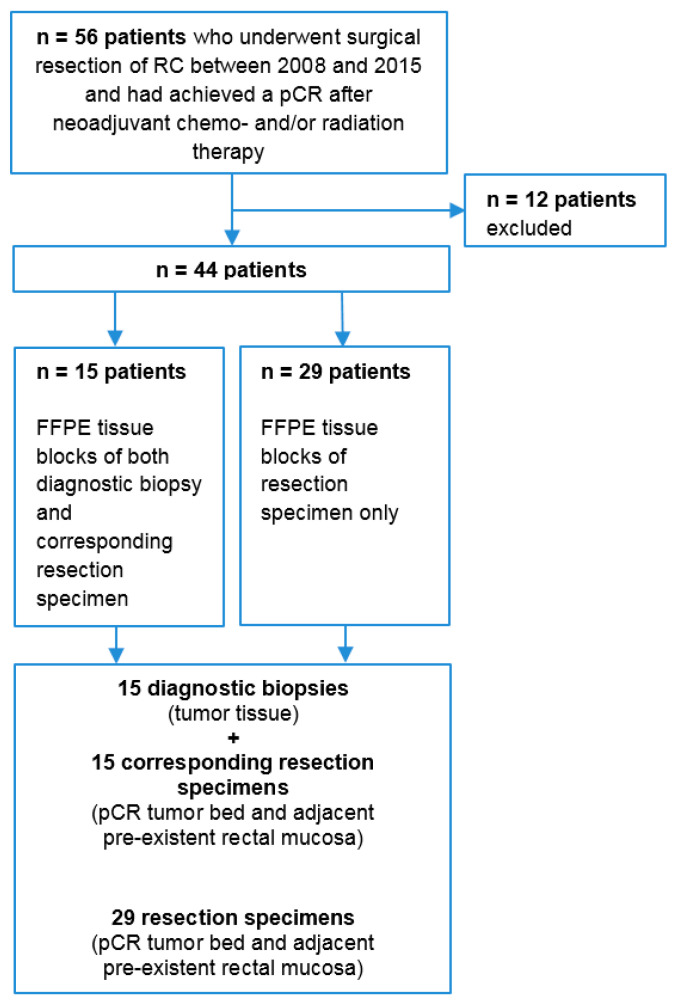
Overview of included tissues. Notes: Described are the number of included tissues. Fifty-six patients were selected. Twelve patients were excluded. From fifteen of the remaining 44 patients, FFPE tissue blocks of both diagnostic biopsy and resection specimen of the corresponding primary tumor were available. From 29 patients, only tissue blocks of the resection specimen were available. Abbreviations: RC, rectal cancer; pCR, pathological complete response; FFPE, formalin-fixed paraffin-embedded.

Available formalin-fixed paraffin-embedded (FFPE) tissue blocks of diagnostic biopsy and resection specimens of the primary tumor from 56 patients who underwent surgical resection of locally advanced rectal cancer between 2008 and 2015 and had achieved a pCR after neoadjuvant chemo- and/or radiation therapy were collected from the Laboratory of Pathology (Stichting Pathology and Medical Microbiology), associated to the Catharina Hospital Eindhoven, The Netherlands (Figure 1).

Twelve of fifty-six patients were excluded because no tissue was available (*n* = 3), there was no pathological complete response upon revision of the pathology (*n* = 5), or tissue was of poor quality (*n* = 4). From 15 of the remaining 44 patients, tissue blocks of both the diagnostic biopsy and the resection specimen of the corresponding primary tumor were available. From the remaining 29 patients, only tissue blocks of the resection specimen were available.

Medical records and pathology reports were retrospectively reviewed. A representative FFPE tissue block of the primary resection specimen of each patient, and the diagnostic biopsy when available, was chosen by a board-certified gastrointestinal pathologist (AFS). Tumor bed was selected as the area in the specimen with reactive changes as a result of the neoadjuvant therapy, mainly ulceration, acellular mucinous pools, and fibrosis. Ideally, the selected slides were representative of the tumor bed in relation to adjacent pre-existent rectal mucosa.

All patients had given informed consent for retrospective use of their archived tissues. All samples were nonidentifiable and used in accordance with the code for proper secondary use of human tissue as prescribed by the Dutch Federation of Medical Scientific Societies and conformed to a protocol that had been reviewed and approved by the institutional review board of the Leiden University Medical Center (LUMC). This study was conducted in accordance with the Declaration of Helsinki. 

### 2.2. Immunohistochemistry (IHC)

After cutting the FFPE blocks into 4 μm sections, these were mounted on adhesive slides (Starfrost, Waldemar Knittel Glasbearbeitungs GmbH, Braunschweig, Germany), deparaffinized using xylene and rehydrated in decreasing concentrations of ethanol. Subsequently, slides were rinsed with demineralized water, and endogenous peroxidase was blocked with 0.3% hydrogen peroxidase (Merck Millipore, Darmstadt, Germany) for 20 min at room temperature. Slides were rinsed with demineralized water, and antigen retrieval was performed in the PT Link (Agilent, Santa Clara, CA, USA), using either Target Retrieval Solution pH 6.0 (Agilent, Santa Clara, CA, USA) at 95 °C or 0.4% pepsin at 37 °C for 10 min (Table A1). After rinsing with phosphate-buffered saline (PBS, pH 7.4), slides were stained overnight at room temperature with primary antibodies, diluted in 1% bovine serum albumin/PBS, against CEACAM5 (clone CI-P83-1, SantaCruz Biotechnology, 200 μg/mL, dilution 1:1000), EpCAM (clone MOC31, Acris Antibodies, 0.64 mg/mL, dilution 1:10,000), αvβ6 (clone 6.2A1, Biogen Idec, 0.5 μg/mL, not diluted), and uPAR (clone ATN-617, Monopar Therapeutics Inc., 0.48 mg/mL, dilution 1:400). After three PBS washing steps, the slides were incubated with a horseradish peroxidase (HRP)-labelled secondary antibody against mouse (EnVision, Agilent, Santa Clara, USA) for 30 min at room temperature. Binding of the antibody was visualized using 3,3′-diaminobenzidine (Agilent, Santa Clara, USA). All slides were counterstained with hematoxylin for 10–15 s, dehydrated at 37 °C, and mounted with pertex. Slides were scanned using the Philips IntelliSite Pathology Solution (Philips Electronics, Eindhoven, The Netherlands). 

### 2.3. Scoring Method

All diagnostic biopsies and primary resection specimens from patients with rectal cancer who had achieved a pCR after neoadjuvant therapy were scored for expression of CEA, EpCAM, αvβ6, and uPAR. Not all tissues have been scored for all four markers due to incidental poor slide quality. The total immunostaining score (TIS) was calculated by multiplying the proportion score (PS) by the intensity score (IS) [22]. The PS represented the percentage of positively stained cells and ranged between 0 and 4 (0 = none; 1 < 10%; 2 = 10–50%; 3 = 51–80%; 4 > 80%). The IS represented the intensity of the stained cells and could range between 0 and 3 (0 = no staining; 1 = weak; 2 = moderate; 3 = strong). Subgroups were defined based on the calculated TIS: 0, no expression; 1–5, weak expression; 6–8, moderate expression; 9–12, intense expression. For dichotomization of subgroups, TIS 0–5 (no to weak expression) was regarded as low expression, TIS 6–12 (moderate-to-intense expression) as high expression. IHC staining scoring was performed by two independent observers (AFS and WT). The observers were blinded for the origin of the tissues. The weighted Kappa was 0.90. In case of disagreement, the mean of the two observed total immunostaining scores, rounded upwards, was used.

### 2.4. Statistical Analysis

Statistical analyses were performed using SPSS version 23.0 software (SPSS, IBM Corporation, NY, USA) and GraphPad Prism 6 (GraphPad Software Inc., La Jolla, CA, USA). For each patient, differences in expression levels between tumor tissue in the diagnostic biopsy and tumor bed in the corresponding resection specimen were calculated using the Wilcoxon signed-rank test. This test was also used to calculate differences in expression levels between tumor bed and adjacent pre-existent rectal mucosa in the resection specimen per patient. A Kruskal–Wallis test was used to determine the differences in tumor bed-to-pre-existent rectal mucosa protein expression ratio between all four biomarkers. In all tests, results were considered statistically significant at the level of *p* < 0.05. 

## 3. Results

Patient and tumor characteristics are summarized in Table 1. Median time to surgery was 11 weeks. For 36 patients, the neoadjuvant therapy consisted of radiotherapy with a total dose of 50 Gray in 25 fractions in combination with capecitabine. Four patients received short-course radiotherapy with a total dose of 25 Gray in five fractions, followed by an extended waiting period (median time to surgery of these four patients was 18 weeks). Another four patients participated in a clinical trial (RAPIDO trial, NCT01558921) and received radiotherapy with a total dose of 25 Gray in five fractions, followed by a median of six courses capecitabine and oxaliplatin, with or without bevacizumab.

### 3.1. CEA, EpCAM, αvβ6, and uPAR Expression

**Figure 2:**
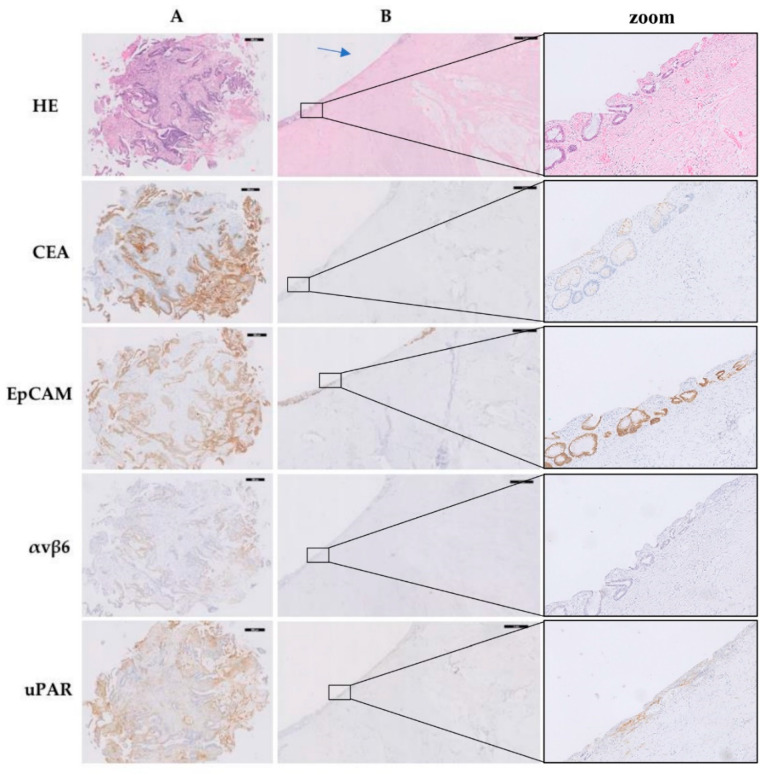
Representative images of CEA, EpCAM, αvβ6, and uPAR expression in a diagnostic biopsy and corresponding primary resection specimen derived from one patient with rectal cancer with a pCR after neoadjuvant therapy. (**A**) Diagnostic biopsy (magnification 5×, black bar = 200 µm). (**B**) Corresponding primary resection specimen (magnification 1×, black bar = 1 mm). The arrow indicates the location of the tumor bed. The zoom contains a magnification of the transition area of tumor bed to adjacent pre-existent rectal mucosa (magnification 20×). Abbreviations: HE, hematoxylin–eosin; CEA, carcinoembryonic antigen; EpCAM, epithelial cell adhesion molecule; αvβ6, integrin αvβ6; uPAR, urokinase-type plasminogen activator receptor; pCR, pathological complete response.

Figure 2 shows representative CEA, EpCAM, αvβ6, and uPAR stained tissue slides of a diagnostic biopsy and corresponding primary resection specimen derived from one patient. On tumor cells (Figure 2A), CEA expression was highest on the apical membrane. EpCAM and αvβ6 showed a membranous, circumferential staining pattern. In the resected primary tumor bed (Figure 2B), target expression was predominantly absent, due to the lack of epithelial cells. In some patients, CEA, EpCAM, and αvβ6 showed nonspecific staining in fibrosis, necrotic areas, and acellular mucin lakes. uPAR was expressed by (cancer-associated) fibroblasts in the diagnostic biopsies and resected primary tumor beds. CEA, αvβ6, and in particular EpCAM were to some extent positive in pre-existent rectal mucosa. 

**Figure 3:**
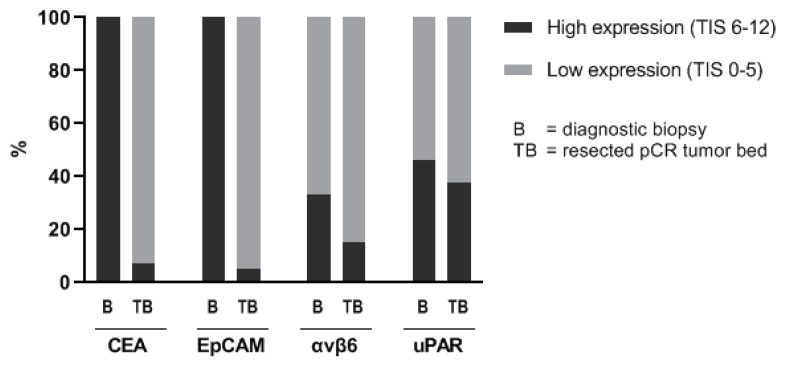
High and low expression in the diagnostic biopsy and resected pCR tumor bed. Notes: Shown are the percentages of diagnostic biopsies and resected pCR tumor beds with a high (TIS 6–12) and low (TIS 0–5) expression of each marker. The total number of scored biopsies and tumor beds was respectively 15 and 40 for CEA, 15 and 39 for EpCAM, 15 and 40 for αvβ6, and 13 and 40 for uPAR. Abbreviations: TIS, total immunostaining score; CEA, carcinoembryonic antigen; EpCAM, epithelial cell adhesion molecule; αvβ6, integrin αvβ6; uPAR, urokinase-type plasminogen activator receptor; pCR, pathologic complete response.

Total immunostaining scores (TIS) of all four targets in all scored tissues are summarized in Table 2. Figure 3 shows the percentages of diagnostic biopsies and resection specimens with a high (TIS 6–12) and low (TIS 0–5) expression of each marker. High TIS was seen in 100% of the diagnostic biopsies for CEA, 100% for EpCAM, 33% for αvβ6, and 46% for uPAR. Conversely, there was low or no expression of CEA in 93% of the resection specimens, of EpCAM in 95%, of αvβ6 in 85%, and of uPAR in 62.5%. In adjacent pre-existent rectal mucosa, defined as healthy rectal mucosa exposed to neoadjuvant chemoradiation therapy, targets had a high expression in 80% (CEA), 95% (EpCAM), 52% (αvβ6), and 3% (uPAR). In Table A2, the number of patients for which all corresponding tissues have been scored are summarized per marker.

### 3.2. Comparison of Protein Expression in Tumor Tissue in Diagnostic Biopsy and Corresponding Tumor Bed after Neoadjuvant Therapy

**Figure 4:**
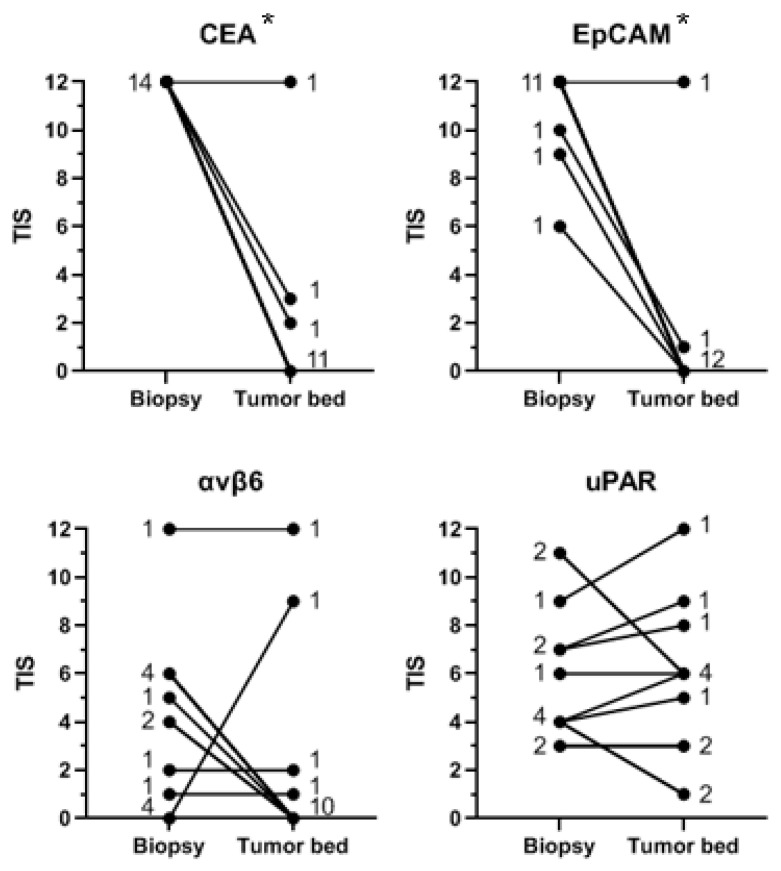
Target expression in tumor tissue in the diagnostic biopsy compared to the pCR tumor bed in the corresponding resection specimen. Notes: Shown is the target expression (as TIS) in the diagnostic biopsy compared to the pCR tumor bed in the corresponding resection specimen, per patient and per target. Every line represents one or more patients. The number of patients with a certain expression score (TIS) in the biopsy and tumor bed is indicated next to the corresponding lines. A horizontal line indicates the same level of expression between biopsy and tumor bed. A descending or ascending line indicates respectively a higher or lower expression in the biopsy compared with the tumor bed. The asterisks (*) indicate a significantly higher expression in the diagnostic biopsy compared with the corresponding pCR tumor bed in the resection specimen. Abbreviations: TIS, total immunostaining score; CEA, carcinoembryonic antigen; EpCAM, epithelial cell adhesion molecule; αvβ6, integrin αvβ6; uPAR, urokinase-type plasminogen activator receptor; pCR, pathological complete response.

From fifteen patients, both the diagnostic biopsy and the corresponding resection specimen were available. Figure 4 shows the TIS of CEA, EpCAM, αvβ6, and uPAR in the diagnostic biopsy compared to the tumor bed in the corresponding resection specimen for each patient. CEA and EpCAM expression were significantly higher in the biopsies than in the tumor bed of the corresponding resection specimens (*p* < 0.01 for CEA and EpCAM). αvβ6 and uPAR expression was not significantly higher in the biopsies (respectively *p* = 0.082 and *p* = 0.246). Median TIS in the diagnostic biopsies was respectively 12 (range 11–12) for CEA, 12 (range 6–12) for EpCAM, 4 (range 0–12) for αvβ6, and 4 (range 3–11) for uPAR. Median TIS in the tumor bed was respectively 0 (range 0–12) for CEA, 0 (range 0–12) for EpCAM, 6 (range 0–12) for αvβ6, and 1 (range 0–12) for uPAR.

### 3.3. Comparison of Protein Expression in Tumor Bed and Adjacent Pre-Existent Rectal Mucosa

**Figure 5:**
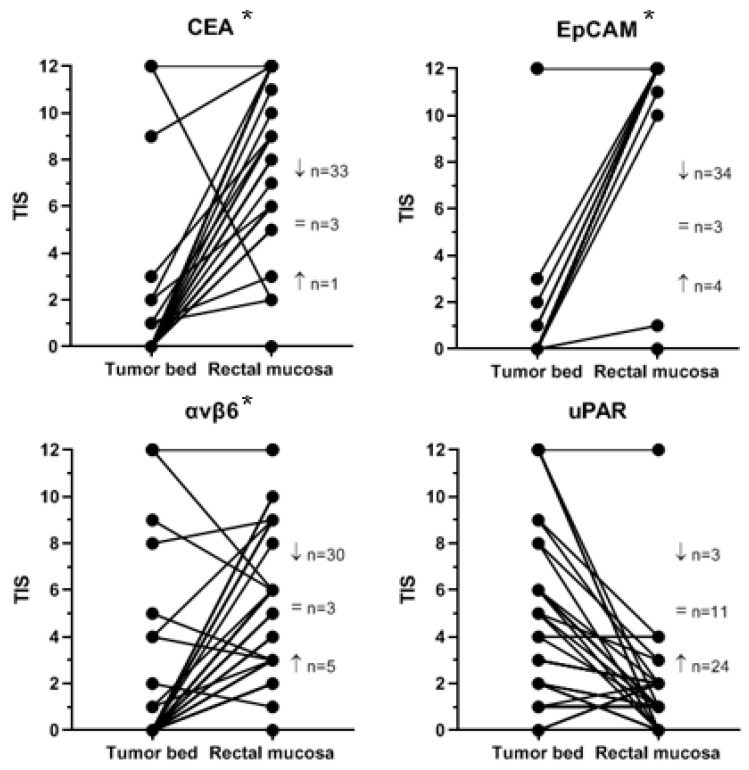
Target expression in the tumor bed compared to adjacent pre-existent rectal mucosa in resection specimens of pathological complete responders. Notes: Shown is the target expression (as TIS) in the pCR tumor bed compared to adjacent pre-existent rectal mucosa in the resection specimen, per patient and per target. Every line represents one or more patients. A horizontal line indicates the same level of expression in tumor bed and adjacent pre-existent mucosa. A descending or ascending line indicates respectively a higher or lower expression in the tumor bed compared with the adjacent pre-existent mucosa. The arrows indicate the number of tumor beds that show a decreased or enhanced expression compared with adjacent pre-existent mucosa; =refers to equal expression scores. The asterisks (*) indicate a significantly lower expression in the tumor bed compared with adjacent pre-existent rectal mucosa. Abbreviations: TIS, total immunostaining score; CEA, carcinoembryonic antigen; EpCAM, epithelial cell adhesion molecule; αvβ6, integrin αvβ6; uPAR, urokinase-type plasminogen activator receptor; pCR, pathological complete response.

Figure 5 shows the TIS of CEA, EpCAM, αvβ6, and uPAR for each patient in the tumor bed compared to adjacent pre-existent rectal mucosa in the resection specimen. CEA, EpCAM, and αvβ6 expression was significantly lower in the tumor bed compared with adjacent pre-existent mucosa (*p* < 0.01 for CEA, EpCAM, and αvβ6). uPAR expression was significantly higher in the tumor bed compared with adjacent pre-existent mucosa (*p* < 0.05). Median TIS in the tumor bed and adjacent pre-existent mucosa was respectively 0 (range 0–12) and 9 (range 0–12) for CEA, 0 (range 0–12) and 12 (range 0–12) for EpCAM, 0 (range 0–12) and 6 (range 0–12) for αvβ6, and 3.5 (range 0–12) and 1 (range 0–12) for uPAR. A significant difference in the tumor bed-to-pre-existent mucosa expression ratio between biomarkers was found, with the lowest rank for EpCAM, followed by CEA, αvβ6, and uPAR (*p* < 0.01).

## 4. Discussion

Response evaluation after neoadjuvant therapy using targeted NIR fluorescence endoscopy has the potential for more accurate selection of rectal cancer patients for a W&W strategy, avoiding both unnecessary surgery and local regrowth. The present study investigated the expression of four promising biomarkers in the diagnostic biopsy, corresponding resected tumor bed, and adjacent pre-existent mucosa of rectal cancer patients with a pCR after neoadjuvant treatment. Our study demonstrates that the targets CEA and EpCAM are absent or have low expression in the tumor bed of nearly all rectal cancer patients with a pCR. Our data also confirm that CEA and EpCAM expression is significantly higher in tumor tissue of the diagnostic biopsy compared with the corresponding resected pCR tumor bed. Furthermore, we show that CEA, EpCAM, and αvβ6 expression is significantly lower in the pCR tumor bed compared with adjacent pre-existent mucosa. In contrast, uPAR is highly expressed in the pCR tumor bed of a significant number of patients and is not upregulated in the diagnostic biopsies compared with the corresponding pCR tumor bed. In addition, uPAR expression is not significantly lower in the pCR tumor bed compared with adjacent pre-existent mucosa. 

Even though the membrane proteins αvβ6 and uPAR are associated with rectal cancer, their relatively high expression in the tumor bed of patients with a pCR makes these biomarkers less suitable as a target for response evaluation. A possible explanation for this high expression in the tumor bed after neoadjuvant therapy is that αvβ6 and uPAR both play an important role in tissue remodeling and wound healing [28,36]. 

Our findings indicate that the cell adhesion molecules CEA and EpCAM could be used as targets for response evaluation using NIR fluorescence endoscopy. A prerequisite for this technique is a tumor target with a significant different expression between tumor and healthy tissue, whose expression is not influenced by neoadjuvant therapy and is absent when there is no residual tumor. Previous research has shown that CEA and EpCAM expression is upregulated in rectal cancer cells compared with adjacent pre-existent rectal mucosa [20,34]. A previous study by our group has demonstrated that CEA and EpCAM are still overexpressed in patients with a partial or no response after neoadjuvant therapy [20,34,35]. In that study, CEA and EpCAM expression after neoadjuvant therapy was high (TIS > 6) in respectively 93% and 100% of the partial- and nonresponders [35]. The current study demonstrates that CEA and EpCAM have an absent or low expression (TIS < 6) in the tumor bed of nearly all patients with a pCR after neoadjuvant therapy. Therefore, both CEA and EpCAM could be suitable targets for response evaluation using fluorescence endoscopy. Both the current and a previous study by our group show that the median CEA expression measured by immunohistochemistry in pre-existent rectal mucosa is lower compared with EpCAM [35]. This would, in theory, favor CEA above EpCAM as a target. However, both studies have been carried out in small groups of patients and might be underpowered to draw this conclusion. 

Although these results are promising, the present study has some limitations. The main drawbacks are the small number of patients from whom both the biopsy and resection specimen were available and the use of semiquantitative IHC to measure protein expression. Validated antibodies and a previously evaluated scoring method were used to minimize variability of the performed IHC [22]. Still, differences in staining intensities between immunohistochemical studies could be observed due to the use of different antibody clones against the same target. Moreover, the degree of correlation between CEA, EpCAM, αvβ6, and uPAR expression measured by IHC in FFPE material and in vivo expression as measured by fluorescence signal intensity has yet to be elucidated in clinical trials. However, clinical trials investigating c-Met-targeted fluorescence endoscopy have demonstrated an excellent correlation between the fluorescence signal intensity measured in vivo and the expression of the target measured with IHC [37,38], indicating the clinical relevance of IHC expression data of potential targets. Although the expression of CEA and EpCAM has already been demonstrated in patients without a pCR after neoadjuvant therapy [35], future studies should include these patients as a control group to directly compare marker expression between patients with and without a pCR.

In the treatment of rectal cancer, the application of targeted fluorescence contrast agents in combination with an endoscope equipped with NIR fluorescence light has the potential to correctly identify patients with a complete response after neoadjuvant therapy. This is illustrated by the fact that fluorescence endoscopy with a topical or intravenously applied contrast agent enables visualization of neoplastic lesions that are visible with conventional endoscopy, as well as additional neoplastic lesions that are missed by conventional endoscopy alone [39,40,41,42]. An ongoing clinical trial investigates the use of vascular endothelial growth factor (VEGF)-targeted fluorescence endoscopy for response evaluation in rectal cancer patients following neoadjuvant therapy (ClinicalTrials.gov Identifier: NCT01972373). A previous pilot study indicated that, like CEA and EpCAM, expression of the rectal cancer-associated membrane protein VEGF is absent in the tumor bed of patients with a pCR. It also demonstrated that VEGF-targeted fluorescence endoscopy has a higher sensitivity to detect residual tumor compared with MRI combined with conventional endoscopy [43]. This improved sensitivity demonstrates the possible value of fluorescence endoscopy for a better identification of residual tumor, decreasing understaging and local regrowth. Furthermore, the expression of CEA and EpCAM, in contrast to VEGF expression, is not influenced by neoadjuvant therapy, possibly making these markers even more suitable for response evaluation [44]. 

Possible limitations of fluorescence endoscopy for response evaluation could be false-positive results due to nonspecific positivity in, for instance, mucin lakes and fibrosis or false-negatives due to complete submucosal localization of residual tumor or isolated metastatic lymph nodes. However, a totally submucosal residual or recurrent tumor after neoadjuvant therapy, with no tumor reaching the mucosa, is rare, occurring in only 1% of cases [45]. Furthermore, NIR light penetrates tissues by up to ~8 mm, probably enabling detection of the vast majority of submucosal-located residual tumors [43,46].

The use of fluorescence-labeled contrast agents targeting CEA and EpCAM has been shown to be safe and feasible for tumor imaging in humans and is the subject of extensive investigation. In colorectal and pancreatic cancer surgery, the intraoperative use of a fluorochrome-labeled anti-CEA monoclonal antibody, SGM-101, provides an enhanced differentiation between tumor and normal tissue [19]. It seems to facilitate the detection of additional neoplastic lesions, changing the final treatment strategy in 35% of colorectal patients [15]. Recently, two phase III randomized controlled trials were initiated, further investigating the intraoperative use of SGM-101 in colorectal cancer surgery (ClinicalTrials.gov Identifier: NCT03659448 and Netherlands Trial Register, ID NL7653). An ongoing phase I clinical dose escalation and optimization trial explores the intraoperative use of an EpCAM-specific fluorescence agent in esophageal, gastric, and rectosigmoid cancer (Netherlands Trial Register, ID NL7363). Since these fluorescence contrast agents against CEA and EpCAM have been introduced in the clinic and have proven to be safe and effective, rapid implementation of these agents in NIR fluorescence endoscopy is possible. However, future trials regarding the clinical application of these markers in NIR fluorescence endoscopy require development of additional, target-specific fluorescence endoscopes and an optimal contrast agent dose. Therefore, future research should focus on dose escalation and the added value of this technique in correctly classifying rectal cancer patients with a complete response after neoadjuvant therapy.

## 5. Conclusions

CEA and EpCAM seem to be suitable targets for response evaluation in rectal cancer using NIR fluorescence endoscopy, since immunohistochemical evaluation shows that expression of these markers in the tumor bed is low compared with the diagnostic biopsy and adjacent pre-existent rectal mucosa in nearly all (>90%) patients with a pCR.

## Figures and Tables

**Table 1:** Patient and tumor characteristics.

	Biopsy and Resection (*n* = 15)	Only Resection (*n* = 29)	All (*n* = 44)
Age at surgery, median years (range)	71 (48–88)	61 (28–78)	66 (28–88)
Gender			
*Male*	10	13	23
*Female*	5	16	21
Tumor type			
*Adenocarcinoma*	14	24	38
*Tubulovillous adenoma with dysplasia*	1	1	2
*Unknown*	0	4	4
Tumor size, median cm (range)	5 (3–12)	5 (2–15)	5 (2–15)
Type of neoadjuvant therapy			
*25 × 2 Gy + capecitabine*	12	24	36
*5 × 5 Gy*	2	2	4
*5 × 5 Gy + capecitabine*, *oxaliplatin*, *bevacizumab*	0	2	2
*5 × 5 Gy + capecitabine*, *oxaliplatin*	1	1	2
Type of surgery			
*Low anterior resection*	7	15	22
*Abdominoperineal resection*	6	12	18
*Transanal endoscopic microsurgery*	1	2	3
*Hartmann’s procedure*	1	0	1
Clinical stage			
*Tumor stage*, *n*			
cTx	5	7	12
cT2	2	1	3
cT3	7	9	16
cT4	1	12	13
*Nodal stage*, *n*			
cNx	5	7	12
cN0	2	5	7
cN1	5	3	8
cN2	3	14	17
*Metastatic stage*, *n*			
cMx	6	7	13
cM0	9	20	29
cM1	0	2	2
Pathologic stage			
*Tumor stage*, *n*			
pT0	15	29	44
*Nodal stage*, *n*			
pN0	14	29	43
pN1	1	0	1
*Metastatic stage*, *n*			
pM0	15	27	42
pM1	0	2	2
Time between neoadjuvant therapy and surgery, median weeks (range)			11 (6–57)

Abbreviations: Gy, Gray; n, number of patients; c, clinical; p, pathological.

**Table 2:** Total immunostaining scores (TIS) of all stained tissues.

Total Immunostaining Score (TIS) on Biopsy, pCR Tumor Bed, and Pre-Existent Rectal Mucosa *n* (%)					
Expression TIS	No 0	Weak 1–5	Moderate 6–8	Intense 9–12	Total Tissues
CEA					
Biopsy	0 (0%)	0 (0%)	0 (0%)	15 (100%)	15
Tumor bed	30 (75%)	7 (18%)	0 (0%)	3 (7%)	40
Pre-existent rectal mucosa	2 (5%)	6 (15%)	10 (25%)	22 (55%)	40
EpCAM					
Biopsy	0 (0%)	0 (0%)	1 (7%)	14 (93%)	15
Tumor bed	33 (85%)	4 (10%)	0 (0%)	2 (5%)	39
Pre-existent rectal mucosa	1 (2.5%)	1 (2.5%)	0 (0%)	39 (95%)	41
αvβ6					
Biopsy	4 (27%)	6 (40%)	4 (27%)	1 (6%)	15
Tumor bed	28 (70%)	6 (15%)	2 (5%)	4 (10%)	40
Pre-existent rectal mucosa	1 (3%)	19 (45%)	11 (26%)	11 (26%)	42
uPAR					
Biopsy	0 (0%)	7 (54%)	3 (23%)	3 (23%)	13
Tumor bed	5 (12.5%)	20 (50%)	8 (20%)	7 (17.5%)	40
Pre-existent rectal mucosa	10 (24%)	30 (73%)	0 (0%)	1 (3%)	41

Notes: Shown are the number (and percentage) of all stained tissues with a certain total immunostaining score (TIS), per target and per tissue type. Subgroups were defined based on the TIS: 0, no expression; 1–5, weak expression; 6–8, moderate expression; 9–12, intense expression. Tissue types were diagnostic biopsy, pCR tumor bed in the resection specimen, and adjacent pre-existent rectal mucosa in the resection specimen. Abbreviations: TIS, total immunostaining score; CEA, carcinoembryonic antigen; EpCAM, epithelial cell adhesion molecule; αvβ6, integrin αvβ6; uPAR, urokinase-type plasminogen activator receptor.

## Data Availability

The data presented in this study are available on request from the corresponding author.

## Appendix A

**Table A1:** Primary antibodies used.

Antibody	Company	Clone	Stock Concentration	Dilution	Antigen Retrieval
Anti-CEACAM5	SantaCruz Biotechnology	Cl-P83-1	0.2 µg/mL	1/1000	Dako PT Link Target Retrieval Solution, pH 6.0
Anti-EpCAM	Acris Antibodies	MOC31	0.64 mg/mL	1/10,000	Dako PT Link Target Retrieval Solution, pH 6.0
Anti-αvβ6	Biogen Idec	6.2A1	0.5 µg/mL	-	0.4% Pepsin
Anti-uPAR	Monopar Therapeutics Inc	ATN617	0.48 mg/mL	1/400	Dako PT Link Target Retrieval Solution, pH 6.0

Abbreviations: CEACAM5, carcinoembryonic antigen-related cell adhesion molecule 5; EpCAM, epithelial cell adhesion molecule; αvβ6, integrin αvβ6; uPAR, urokinase-type plasminogen activator receptor.

**Table A2:** Available stained tissue “pairs” per marker.

	Biopsy and Resection (*n*)	Tumor Bed and Normal Epithelium (*n*)
**CEA**	14	37
**EpCAM**	14	37
**αvβ6**	14	38
**uPAR**	12	38

Notes: Not all tissues have been scored for all four markers due to incidental poor slide quality. Therefore, not all fifteen biopsies had a corresponding resection specimen for every marker, and not all 44 resection specimens had both tumor bed and pre-existent rectal mucosa scored. Listed are the number of patients per marker of which both a biopsy and primary resection specimen or both tumor bed and pre-existent mucosa in the resection specimen were scored. Abbreviations: CEA, carcinoembryonic antigen; EpCAM, epithelial cell adhesion molecule; αvβ6, integrin αvβ6; uPAR, urokinase-type plasminogen activator receptor; n, number of patients.
